# Is there a difference in venous thrombosis rate in free flap anastomoses based on coupler diameter? A systematic review. Does Size Really Matter?

**DOI:** 10.1016/j.jpra.2021.07.005

**Published:** 2021-08-11

**Authors:** D.D. Krijgh, B. Tellier, T. Teunis, W. Maarse, J.H. Coert

**Affiliations:** Department of Plastic and Reconstructive Surgery, University Medical Center Utrecht, The Netherlands

**Keywords:** Thrombosis, Anastomotic Coupler Device, Microsurgery

## Abstract

**Background:**

The adage is to use the largest anastomotic coupler device (coupler) size possible, since smaller an anastomosis might be more susceptible to thrombosis. It is unclear if this wisdom is supported by data. This study tests the hypothesis that there is no difference in the reported literature in thrombosis rate between different coupler sizes.

**Methods:**

We searched PubMed, Embase, and the Cochrane Library. After screening 235 studies, we included 11 retrospective case-series. According to the criteria of Newcastle–Ottowa Scale, quality score ranged from 2 to 4 (out of 5) and funnel plots indicated publication bias. We included a total of 5930 coupled anastomoses. We calculated thrombosis rate per coupler diameter with exact confidence intervals (CIs). We regard non-overlapping CIs as a significant difference.

**Results:**

Nine studies reported no difference in thrombosis rate based on coupler size. Two studies report a potentially greater thrombosis rates in smaller sizes: (1) 2.0 mm 27% (95% CI 17%–40%, 17/62 cases) vs. 3.0 mm 6.3% (95% CI 2.8%–12%, 8/126 cases) and (2) 1.5 mm 6.9% (95% CI 2.8%–14%, 7/101 cases) vs. 3.0 mm group 1.2% (95% CI 0.64%–2.1%, 13/1079).

**Conclusion:**

There is some evidence that suggests that smaller coupler sizes are associated with greater thrombosis rate, but the current available evidence has limitations. Performing a second anastomosis, in case, the first anastomosis is performed with a coupler size of 1.0, 1.5, or even 2.0 mm, can potentially reduce this rate, however, this remains to be determined.

## Introduction

The number of free tissue transfers performed by reconstructive surgeons has increased over the last decades.^1^ Many advances have been made to reduce complications and operation time. One of these advances is the anastomotic coupler device (or simply referred to as “coupler”) to simplify the process of a microsurgical anastomosis. Already designed and tested in the early 1960s, the coupler only became widely used over the last two decades.^2^ Although it is possible to use the coupler for an arterial anastomosis, most microsurgeons prefer to use it for a venous anastomosis due to the fact that the venous vessel wall is thinner and, therefore, it is easily everted and pinned to the device.^3^

The couplers used in microsurgery are available in sizes of 1.0 mm–4.0 mm. The adage is to use the largest size possible, as permitted by the vessel with the smallest diameter. Based on the law of Hagen–Poiseuille coupler diameter might not matter. The flowrate is proportional to the radius to the fourth power and if the radius of a lumen decreases, the pressure has to increase to restore the flowrate to normal. Within the human body vasodilation and an increase in blood pressure can only partially restore the flowrate. Furthermore, the law of Hagen–Poiseuille requires perfect laminar flow. In other words, there should be no turbulence. In a microvascular anastomosis, there are always some vessel wall irregularities, and some turbulence can be expected, leading to the possible formation of a blood clot. Because of these irregularities and because of a lower flowrate, smaller vessels potentially become more quickly occluded by a clot. We are interested if the wisdom of needing to use the largest coupler size possible can be verified by actual data. If smaller coupler sizes lead to larger thrombosis rates, surgeons can opt to perform a second anastomosis. This might reduce flap revision rate and flap loss. However, if there is no difference in thrombosis rates, we can save time by just performing a single anastomosis, even with a smaller diameter coupler. This study tests the primary null hypothesis that there is no difference in the reported literature in thrombosis rate between different coupler sizes. Additionally, we assessed total and partial flap loss rate in the included studies.

## Methods

This study is reported in accordance with the Preferred Reporting Items for Systematic Reviews and Meta-Analyses (PRISMA) statement.^4^ The study has been registered at PROSPERO.

### Literature search

**Figure 1:**
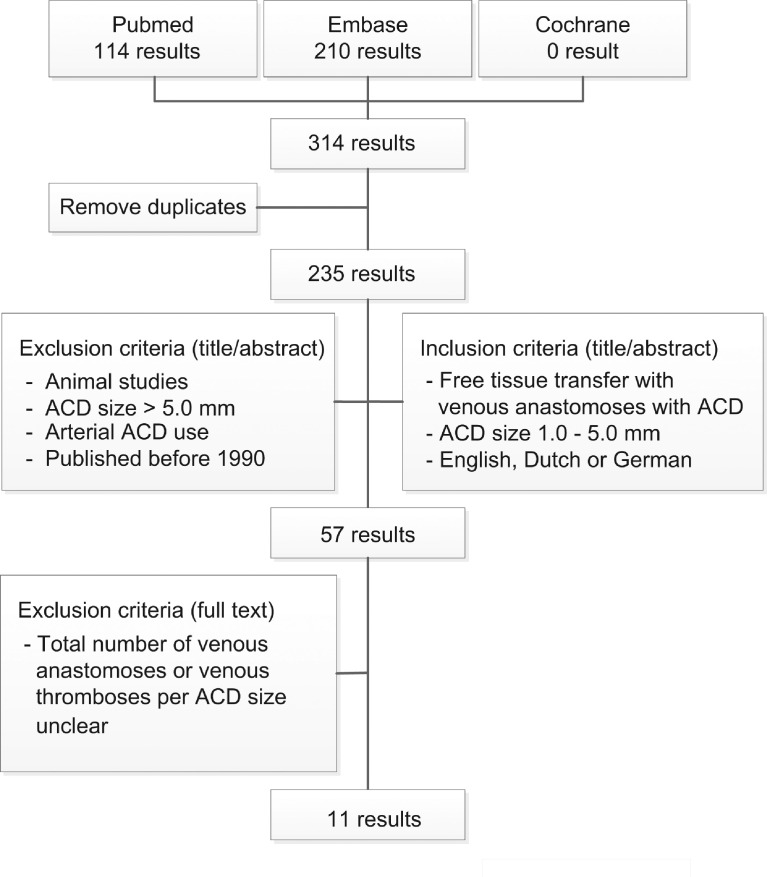
Flowchart systematic literature search Flowchart of literature search in PubMed, Embase, and Cochrane, performed on April 27, 2020.

On April 27, 2020 we searched PubMed, Embase, and the Cochrane Library using search terms based on anastomosis, coupler, or suture. See Appendix 1 for our exact search. After removing duplicates, we found a total of 235 published studies. Articles were screened for relevance by two independent researchers (DDK and BT), using the pre-conceived inclusion and exclusion criteria, as listed in Figure 1. After screening for eligibility, 57 full-text articles were assessed and a total of 11 articles were identified for final inclusion for this systematic review. We searched the references and found no additional articles relevant to this review.

### Data extraction

Two independent reviewers extracted the data. Differences were resolved by discussion until consensus was reached. The following data were extracted using a standardized form: title, authors, year of publication, study design, time period, institution, specialty, anatomic region, pre-operative radiotherapy, sample size, coupler manufacturer, type of reconstruction, venous thrombosis, flap failure rate, partial flap loss, number of free flaps, and incidence of double venous anastomosis.

### Quality assessment

Two reviewers (DDK and TT) together critically appraised the included studies using the five relevant criteria of Newcastle–Ottowa Scale for scoring quality of case-series for systematic reviews.^5^ The articles were assessed for their quality in terms selection and outcome. We regarded the cohort as representative when it included at least 20% oncologic reconstructions and 20% or more traumatic reconstructions.

### Study characteristics

All articles included were retrospective database studies performed between 1990 and 2015.^6^, ^7^, ^8^, ^9^, ^10^, ^11^, ^12^, ^13^, ^14^, ^15^, ^16^ One study consisted of a mix of traumatic and oncologic reconstructions, 4 studies included head and neck reconstructions, 4 studies focused on breast reconstruction, 1 study was a mix of oncological reconstructions, and 1 study consisted solely of lower extremity reconstructions. Two coupler systems by two different producers were included: the Global Excellence in Microsurgery (GEM) microvascular anastomotic coupler system by Synovis (Synovis Micro Companies Alliance, Birmingham, AL) and its precursor, the 3M Unilink System (3M Healthcare, St. Paul, MN; Table 1).

**Table 1:** Overview of included studies.

Article author	Kisser	Hanson	Kulkarni	Wang	Medina	Broer	Jandali	Rad	Shindo	Delacure	Sasson
Year of study	2018	2017	2016	2015	2014	2013	2010	2008	1996	1995	1994
Study design	Retrospective	Retrospective	Retrospective	Retrospective	Retrospective	Retrospective	Retrospective	Retrospective	Retrospective	Retrospective	Retrospective
Time period	2009–2015	2001–2013	1997–2012	2013–2014	2007–2009	2000–2011	2002–2008	2006–2007	1992–1995	1990–1994	1991–1993
Anatomic region	Head & Neck	Mix	Breast	Head & Neck	Lower extremity	Breast	Breast	Breast	Head & Neck	Head & Neck	Mix
Head and neck reconstruction	100%	55,00%		100%					100%	100%	30%
Breast reconstruction		40,00%	100%			100%	100%	100%			
extremity/pelvis/other		5.1%			100%						70%
Radiotherapy pre-operative	80	1135*	206*	unknown	unknown	26	unknown	1	24	5	unknown
Patients (total)	437	4662	647	64	48	197	730	9	76	29	10
Free flaps (total)	437	5643	857	64	49	392	1000	9	79	29	10
Double venous anastomosis	0	0	0	29	0	0	15	1	11	1	0
Coupled anastomoses	437	3257	554	80	48	392	1000	10	105	37	10
Of which arterial	0	0	0	7 (excluded)	0	0	0	0	17 (excluded)	7 (included)	0
Type of coupler used	Synovis	3M/Synovis	Synovis	Synovis	Synovis	Synovis	3M/Synovis	Synovis	3M/Synovis	3M/Synovis	3M/Synovis

⁎ Number of patients who received radiotherapy pre-operative of the patients in the coupler group.

Quality score ranged from 2 to 4 (maximum possible score 5). Eight studies scored 3 points. All studies, except 1, were regarded as unrepresentative due to inadequate case mix. None of the studies performed or mentioned to perform a blinded outcome assessment (Table 2).

**Table 2:** Adapted Newcastle–Ottowa Scale for the assessment of the quality of non-randomized studies in meta-analyses.

Article author	Kisser	Hanson	Kulkarni	Wang	Medina	Broer	Jandali	Rad	Shindo	Delacure	Sasson
**Selection**											
Representative cohort? (20% or more oncology and trauma)	ND	ND	ND	ND	ND	ND	ND	ND	ND	ND	*
Ascertainment of the exposure through records?	*	*	*	ND	*	*	*	*	ND	*	*
**Outcome**											
Blinded assessment of the outcome?	ND	ND	ND	ND	ND	ND	ND	ND	ND	ND	ND
Long enough follow-up?	*	*	*	*	*	*	*	*	*	*	*
Follow-up of the complete cohort?	*	*	*	*	*	*	*	*	*	*	*
Total	3	3	3	2	3	3	3	3	2	3	4

ND= no description

**Figure 2:**
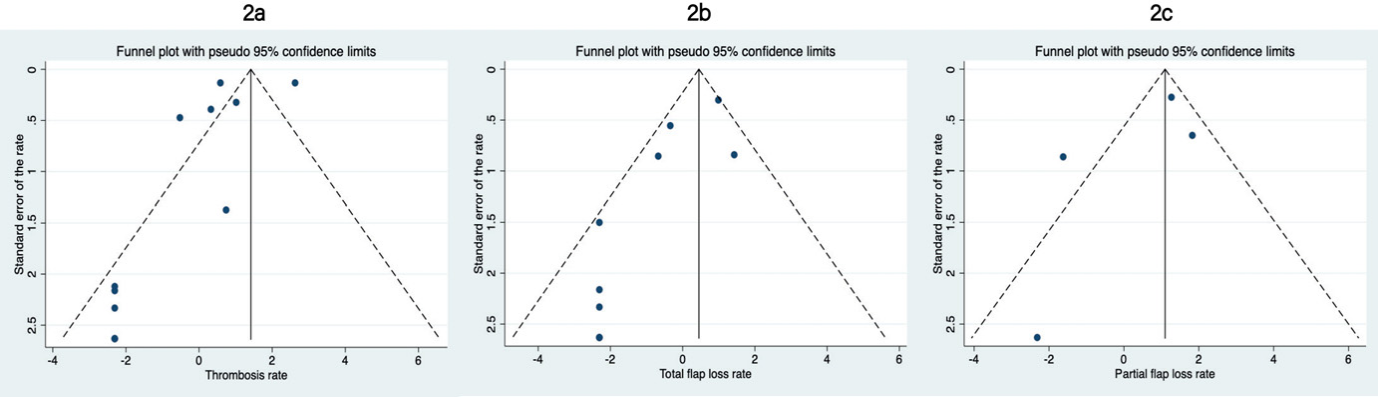
Funnel plots.

All funnel plots indicate publication bias since the bottom right side of the funnel is empty (larger thrombosis, total and partial flap failure rate; Figure 2a, b, and c).

### Study population

For this review, a total of 6472 patients were included. Most studies included patients with previous radiotherapy; this was unknown in 4 studies. Several studies included multiple free flaps in a single patient, leading to a total of 8569 flaps. Five studies included multiple venous anastomoses in a single flap (n=57). These studies did not provide a specific indication for performing a second anastomosis. This resulted in a total inclusion of 5930 coupled anastomoses, of which 3257 (55%) were performed in a single study.^6^ Kisser et al. potentially had a greater thrombosis rate than most other studies: 14% (95% confidence interval (CI) 10%–17%).^14^ There were 3 studies that included arterial anastomoses. In 2 of these studies, it was possible to exclude these arterial anastomoses from our analysis (Table 1).

### Analysis

If we encountered sufficient prospective studies, accounting for patient characteristics, we aimed to perform a meta-analysis. However, since we only were able to include retrospective studies, and the subsequent sensitivity to bias, we narratively report our results. We calculated exact CIs because of the low event rate. In the case of 0 events, we calculated a one-sided 97.5% CI. The cases in our review are not completely independent, due to the fact that we were unable to parse out multiple flaps belonging to the same patient, and flaps with a double venous anastomosis. This violates the assumption of most common statistical tests, and the reliability of the reported event rates and CIs. We regard non-overlapping CIs as an indication of a potential significant difference. We created funnel plots by replacing 0 events with 0.1 event, and the lower 95% CI limit of 0 with 0.001; this results in statistically negative events rates in the plots.

## Results

In a total of 5930 coupled venous anastomoses, 157 thrombosis occurred (2.6%, 95% CI 2.3%–3.1%), range 0% (97.5% CI 0%–4.1%) to 14% (95% CI 10%–17%). The majority of the anastomoses were performed with a 2.5 mm (n=1814, 30.7%) and a 3.0 mm (n=2482, 42.0%) coupler. In only 2.6% (n=152), a 1.0 or 1.5 coupler was used. We identified 2 studies with a potentially greater thrombosis rates in smaller coupler size. Kisser et al. had a thrombosis rate of 17 out of 62 cases in the 2.0 mm coupler group (27%, 95% CI 17%–40%) compared with 8 out of 126 cases in the 3.0 mm group (6.3%, 95% CI 2.8%–12%).^14^ Hanson et al. had a thrombosis rate of 7 out of 101 patients in the 1.5 mm group (6.9%, 95% CI 2.8%–14%) and 13 out of 1079 patients in the 3.0 mm group (1.2%, 95% CI 0.64%–2.1%).^6^ We found no within-groups difference in the other studies (Table 3).

**Table 3:** Results. Number of venous thromboses per coupler size and the calculated total thrombosis rate per coupler size with the calculates, 95% CI.

Article author	N	Kisser	Hanson	Kulkarni	Wang	Medina	Broer	Jandali	Rad	Shindo	Delacure	Sasson
Coupler sizes and thrombosis rate												
**1.0 mm**	5	0/5	NU	NU	NU	NU	NU	NU	NU	NU	NU	NU
Rate (95% CI)		0% (0%–52%)										
**1.5 mm**	147	4/26	7/101	NU	0/8	NU	0/1	0/1	NU	0/9 (0%–34%)	0/1 (0%–98%)	NU
Rate (95% CI)		15% (4.3%–35%)	6.9% (2.8%–14%)	0% (0% to 37%)	0% (0%–98%)	0% (0%–98%)		0%	0%			
**2.0 mm**	585	17/62	5/329	0/46	0/15	NU	5/75	0/14	0/6	0/26	0/12	NU
Rate (95% CI)		27% (17%–40%)	1.3% (0.42%–3.0%)	0% (0%–7.7%)	0% (0%–22%)	6.7% (2.2%––15%)	0% (0%–23%)	0% (0%–46%)	0% (0%–13%)	0% (0%–26%)		
**2.5 mm**	1814	16/142	18/991	4/211	0/20	0/12	5/218	1/130	0/3	0/53	0/24 (0%–14%)	0/10
Rate (95% CI)		11% (6.6%–18%)	1.8% (1.1%–2.9%)	1.9% (0.52%–4.8%)	0% (0%–17%	0% (0%––26%)	2.3% (0.7%––5.3%)	0.77% (0.019%–4.2%)	0% (0%–71%)	0% (0%–6.7%)	0%	0% (0%–31%)
**3.0 mm**	2482	8/126	13/1079	3/278	0/20	1/36	1/90	5/852	0/1	NU	NU	NU
Rate (95% CI)		6.3% (2.8%–12%)	1.2% (0.64%–2.1%)	1.1% (0.22%–3.1%)	0% (0%–17%)	2.8% (0.07%––15%)	1.1% (0.03%–– 6.0%)	0.59% (0.19%–1.4%)	0% (0%–98%)			
**3.5 mm**	587	8/50	12/502	1/19	0/6	NU	0/8	0/2	NU	NU	NU	NU
Rate (95% CI)		16% (7.2%–29%)	2.4% (1.2%–4.1%)	5.3% (0.13%–26%)	0% (0%–46%)	0% (0%––37%)	0% (0%–84%)					
4.0 mm	286	6/26	4/255	NU	0/4	NU	NU	0/1	NU	NU	NU	NU
Rate (95% CI)		23% (9.0%–44%)	1.6% (0.43%–4.0%)	0% (0%–60%		0% (0%–98%)						
**Total**		59/437	60/3257	8/554	0/73	1/48	11/392	6/1000	0/10	0/88	0/37	0/10
Rate (95% CI)		14% (10%–17%)	1.8% (1.4%–2.4%)	1.4% (0.6%–2.8%)	0% (0%–4.9%)	2.1% (0.05%––11%)	2.8% (1.4%––5.0%)	0.6% (0.2%–1.3%)	0% (0%–31%)	0% (0%–4.1%)	0% (0%–9.5%)	0% (0%–31%)
Full flap loss		12	not reported	4	0	2	2	0	0	not reported	0	0
Rate (95% CI)		2.7% (1.4%–4.7%)	0.72% (0.20%–1.8%)	0% (0%–4.9%)	4.2% (0.51%–14%)	0.51% (0.062%––1.8%)	0% (0%–0.37%)	0% (0%–31%)	0% (0%–9.5%)	0% (0%–31%)		
Partial flap loss		not reported	not reported	not reported	not reported	3	14	2	not reported	not reported	not reported	0
Rate (95% CI)						6.3% (1.3%––17%)	3.6% (2.0%––5.9%)	0.20% (0.024%–0.72%)			0% (0%–31%)	

*NU = not used. In case of 0 events, the 95% CI actually is a one-sided 97.5% CI

Nine out of 11 studies reported their full flap loss totaling to 20 out of 2585 patients (0.77%, 95% CI 0.47%–1.2%). Partial flap loss was reported in 4 studies and occurred in total in 19 patients out of 1450 patients (1.3%, 95% CI 0.79%–2.0%). Jandali et al. reported a lower full flap loss rate of 0 out of 1000 (0%, 97.5% CI 0%–0.37%) as well as a lower partial flap loss rate of 2 out of 1000 (0.20%, 95% CI 0.024%–0.72%), compared with most other studies.^7^

## Discussion

When performing a venous anastomosis with an anastomotic coupler device, or “coupler,” the adage is to use the largest coupler size possible, as permitted by the vessel with the smallest diameter. Possibly because of turbulence at the site of the anastomosis blood clots can form, that can subsequently lead to a thrombosis with smaller diameter vessels at a higher risk as compared with larger vessels. We searched the existing literature for evidence supporting or disputing this adage.

Several limitations need to be kept in mind when interpreting the results. First, we were only able to include studies of limited quality. All studies were retrospective, and only a single study appropriately accounted for multiple anastomoses belonging to a single patient and differences in baseline characteristics like previous radiotherapy.^6^ Therefore, it is not appropriate to report aggregate results. This also limits the validity of the CIs we report. Second, there is evidence of publication bias. Funnel plots indicate that particularly smaller studies with a greater thrombosis rate are underrepresented. Our results are likely to be more optimistic (i.e. lower thrombosis, full flap loss, and partial flap loss rates) than can be expected in clinical practice. The included articles in this review are very heterogeneous in the number of patients that were included. Studies that are performed in centers that perform a high number of microsurgical interventions seem to have a lower venous failure rate. Third, only 152 anastomoses (2.6%) were performed with a 1.0 and 1.5 mm coupler. This limits the generalizability of any results found in these groups. Lastly, most of the included articles reported that they only included free flaps with a single venous anastomosis. However, in 5 articles, we were not able to differentiate between single and double venous anastomoses and, therefore, 57 (0.67%) free flaps of the total 8569 free flaps included in our systematic review have had a double venous anastomosis.

We found two studies that suggest a greater venous thrombosis rate in smaller coupler sizes, specifically Kisser et al. (2.0 mm vs. 3.0 mm) and Hanson et al. (1.5 mm vs. 3.0 mm).^6^^,^^14^ The study by Kisser et al. differs from most other studies because it found a high thrombosis rate (14%, 95% CI 10%–17%) compared with other studies (range 0%–2.8%). The study by Hanson et al. is the largest and makes up over half the anastomoses included in this review (n=3257, 55%). They also accounted for multiple flaps belonging to the same patient, and differences in baseline characteristics by generalized estimation equation. Their analysis confirmed the greater thrombosis rate in 1.5 mm couplers compared with 3.0 mm. Although the quality of the aggregate evidence is limited, a venous thrombosis of a free flap anastomosis has major consequences, like unscheduled emergency revisions and partial or total flap loss. To be on the safer side, surgeons can consider additional measures when performing a 1.0, 1.5, or even a 2.0 mm coupler anastomosis. Depending on the situation there are several possibilities: (1) performing a second anastomosis, (2) trim the vein to a more proximal larger caliber, and (3) use an interposition vein graft. Of these possibilities, only the impact of a second venous anastomosis has been studied and is potentially associated with a lower risk for thrombosis and flap failure rate.^17^ Ahmadi et al. conducted a meta-analysis of non-randomized controlled trials with large heterogeneity measured by I^2^ and assessed venous thrombosis rate (14 studies, I^2^ = 51%) and flap failure rates (15 studies, I^2^ = 54%). They report a relative risk reduction of borderline statistical significance in venous thrombosis rate of 0.66 (95% CI 0.46–0.97) and flap failure rate of 0.64 (95% CI 0.41–0.99) when using two venous anastomoses compared with using a single anastomosis. They do not report aggregate absolute risks. Future study could assess the effect of any of the four aforementioned interventions specifically for small caliber coupler anastomosis.

Reported full flap loss ranged from 0% to 4.2% and partial flap loss from 0% to 6.3%. This is similar to what is reported in larger studies that do not specifically look at coupler diameter.^18^ Las et al. reported in 1530 patients with a full flap loss rate of 4.4% (95% CI 3.4–5.5) and a partial flap loss rate of 5.5% (95% CI 4.4–6.8).^19^ Interestingly, we found clear evidence of publication bias (i.e., favoring the publication of positive results over negative results). Smaller studies reporting greater total or partial flap loss are missing from the funnel plots. It is unclear to what extent plastic surgery literature is affected by publication bias. One study found that 77% (98/128) of the abstracts presented at a 2003 and 2004 plastic surgery meeting reported a difference favoring a new intervention.^19^ Future research could study the extent of publication bias in plastic surgery. Authors performing reviews on free flap anastomoses need to be aware of potential publication bias, since this limits generalizability of their results. Descriptive and aggregate results are likely to report lower full and partial flap loss than actually can be expected. Furthermore, it is important to note that articles that have compared a coupled anastomosis to a handsewn anastomosis are very difficult to interpret due to the lack of vessel diameters reported in handsewn anastomoses. Our study shows that a smaller vessel lumen does seem to increase the risk for venous failure and, therefore, it is crucial that future microsurgical research projects do include information about the vessel sizes in handsewn anastomoses.

## Conclusion

A single large study conducting an appropriate analysis suggests that there is a greater thrombosis rate in anastomoses performed with a 1.5 mm coupler compared with a 3.0 mm diameter. Because of the major consequences of a venous thrombosis in a free flap anastomosis, surgeons can consider additional measures, like performing a second anastomosis, in case, the first anastomosis is performed with a coupler size of 1.0, 1.5, or even 2.0 mm. However, if this actually reduces venous anastomosis thrombosis rate, and full or partial flap loss remains to be determined.

## Declaration of Competing Interest

No conflicts of interest

## Appendix Supplementary materials

Image, application 1
