# Supplementary material for: Is there a difference in venous thrombosis rate in free flap anastomoses based on coupler diameter? A systematic review. Does Size Really Matter?
Source: JPRAS Open. 2021 Aug 11;30:74–83. doi: 10.1016/j.jpra.2021.07.005 (PMC8408549; doi:10.1016/j.jpra.2021.07.005)
Supplement: Supplementary file 1 [file mmc1.docx]

**Appendix 1.** Search strategy.

| **PubMed** |
| --- |
| ("Anastomosis, Surgical"[Mesh] OR surgical flaps[MeSH] OR flap[Title/Abstract] OR flaps[Title] OR anastomoses[Title/Abstract] OR anastomosis[Title/Abstract] OR anastomotic[Title/Abstract] OR microanastomosis[Title/Abstract] OR microanastomoses[Title/Abstract] OR micro anastomos*[Title/Abstract]) AND (coupler[Title/Abstract] OR couplers[Title/Abstract] OR coupling[Title/Abstract] OR coupled[Title/Abstract]) AND (handsewn[Title/Abstract] OR hand-sewn[Title/Abstract] OR sutured[Title/Abstract] OR suturing[Title/Abstract] OR sutures[MeSH] OR suture[Title/Abstract] OR sutures[Title/Abstract] OR resutured[Title/Abstract] OR resuturing[Title/Abstract] OR sutures[MeSH] OR resuture[Title/Abstract] OR resutures[Title/Abstract] OR re sutured[Title/Abstract] OR re suturing[Title/Abstract] OR sutures[MeSH] OR re suture[Title/Abstract] OR re sutures[Title/Abstract]) |
| **Embase** |
| “anastomosis”/exp OR “surgical flaps”/exp OR flap:ti,ab,kw OR flaps:ti,ab,kw OR anastomoses:ti,ab,kw OR anastomosis:ti,ab,kw OR anastomotic:ti,ab,kw OR microanastomosis:ti,ab,kw OR microanastomoses:ti,ab,kw OR (‘micro anastomosed’:ti,ab,kw OR “micro anastomoses”:ti,ab,kw OR “micro anastomosis”:ti,ab,kw) AND (coupler:ti,ab,kw OR couplers:ti,ab,kw OR coupling:ti,ab,kw OR coupled:ti,ab,kw OR “vascular closure device”/exp OR “Vascular Closure Devices”:ti,ab,kw OR “Vascular Closure Device”:ti,ab,kw) AND(handsewn:ti,ab,kw OR hand-sewn:ti,ab,kw OR sutured:ti,ab,kw OR suturing:ti,ab,kw OR “suture”/exp OR suture:ti,ab,kw OR sutures:ti,ab,kw OR resutured:ti,ab,kw OR resuturing:ti,ab,kw OR resuture:ti,ab,kw OR resutures:ti,ab,kw OR “re sutured”:ti,ab,kw OR “re suturing”:ti,ab,kw OR “re suture”:ti,ab,kw OR “re sutures”:ti,ab,kw) |
| **COCHRANE** |
| Anastomosis, Surgical; Surgical Anastomosis; Anastomoses, Surgical; Surgical Anastomoses in All Text AND Free Tissue Flaps; Flaps, Microsurgical Free; Free Tissue Transfer Flaps; Tissue Flap, Free; Microsurgical Free Flap; Tissue Flaps, Free; Free Flaps, Microsurgical; Flap, Free; Flap, Microsurgical Free; Microsurgical Free Flaps; Free Flap, Microsurgical; Free Flaps; Free Flap; Flap, Free Tissue; Flaps, Free Tissue; Flaps, Free; Free Tissue Flap; Perforator Flaps; Flaps, Perforator; Flap, Perforator; Island Flaps; Pedicled Flaps; Flap, Pedicled; Pedicled Flap; Flaps, Pedicled; Surgical Flap; Flap, Surgical; Flaps, Surgical in All Text—in Cochrane Reviews, Cochrane Protocols, Trials (Word variations have been searched) |
